# Circulating antioxidants and Alzheimer disease prevention: a Mendelian randomization study

**DOI:** 10.1093/ajcn/nqy225

**Published:** 2018-12-29

**Authors:** Dylan M Williams, Sara Hägg, Nancy L Pedersen

**Affiliations:** 1 Department of Medical Epidemiology and Biostatistics, Karolinska Institute, Stockholm, Sweden; 2 Department of Psychology, University of Southern California, Los Angeles, CA

**Keywords:** vitamin A, vitamin C, urate, uric acid, β-carotene, Alzheimer disease, Mendelian randomization

## Abstract

**Background:**

Higher circulating antioxidant concentrations are associated with a lower risk of late-onset Alzheimer disease (AD) in observational studies, suggesting that diet-sourced antioxidants may be modifiable targets for reducing disease risk. However, observational evidence is prone to substantial biases that limit causal inference, including residual confounding and reverse causation.

**Objectives:**

In order to infer whether long-term circulating antioxidant exposure plays a role in AD etiology, we tested the hypothesis that AD risk would be lower in individuals with lifelong, genetically predicted increases in concentrations of 4 circulating antioxidants that are modifiable by diet.

**Methods:**

Two-sample Mendelian randomization analyses were conducted. First, published genetic association studies were used to identify single-nucleotide polymorphisms (SNPs) that determine variation in circulating ascorbate (vitamin C), β-carotene, retinol (vitamin A), and urate. Second, for each set of SNP data, statistics for genotype associations with AD risk were extracted from data of a genome-wide association study of late-onset AD cases and controls (*n* = 17,008 and 37,154, respectively). Ratio-of-coefficients and inverse-variance-weighted meta-analyses were the primary methods used to assess the 4 sets of SNP-exposure and SNP-AD associations. Additional analyses assessed the potential impact of bias from pleiotropy on estimates.

**Results:**

The models suggested that genetically determined differences in circulating ascorbate, retinol, and urate are not associated with differences in AD risk. All estimates were close to the null, with all ORs for AD ≥1 per unit increase in antioxidant exposure (ranging from 1.00 for ascorbate to 1.05 for retinol). There was little evidence to imply that pleiotropy had biased results.

**Conclusions:**

Our findings suggest that higher exposure to ascorbate, β-carotene, retinol, or urate does not lower the risk of AD. Replication Mendelian randomization studies could assess this further, providing larger AD case-control samples and, ideally, using additional variants to instrument each exposure.

## Introduction

Late-onset Alzheimer disease (AD), the most common form of dementia, is likely to be determined by a combination of inherited genetic risk and environmental influences (1). Identifying modifiable, environmental determinants of AD is crucial for informing public health policies to reduce disease burden in populations.

Lifelong exposure to oxidative stress is hypothesized to hasten neurodegeneration via chronic damage to DNA, lipids, and proteins by oxidation/peroxidation (2). This process could influence AD etiology prior to the development of the pathologic hallmarks of the disease, such as the formation of β-amyloid plaques and neurofibrillary tangles (3). Assuming that the mitigation of oxidative stress would lead to neuroprotection, increases in dietary-sourced and endogenous antioxidants that scavenge free radicals have been proposed as a mechanism to prevent AD onset or to slow its progression (2, 3). Consistent with this hypothesis, case-control and prospective epidemiologic studies have found evidence for lower AD risk in individuals with exposure to higher circulating concentrations of antioxidants (vitamin C, molecules in the vitamin A and E families, and uric acid/urate) or proxy measures of higher exposure (4–10). However, being observational in design, these studies are prone to substantial biases that limit causal inference, including residual confounding and reverse causation (the disease process or its effects on health-related behaviors have affected antioxidant concentrations).

Randomized controlled trials (RCTs) could help to establish the effects of circulating antioxidant modification on cognitive decline and AD risk or progression, but trial data for each exposure are unavailable, scant, or inconclusive at present (11–14). There are several reasons why past trials aimed at AD treatment may have provided null findings, including interventions that target the disease process too late in its development for efficacy (where secondary prevention might be more promising) (15). Moreover, novel trials for the primary prevention of AD would be particularly challenging to conduct, given that the pathogenesis of the disease appears to be perhaps decades in length (16). Other study designs would therefore be valuable for clarifying the role of long-term antioxidant modification in AD etiology. The aim of this study was to examine, through the use of a Mendelian randomization (MR) design (17, 18), whether genetically predicted differences in several circulating antioxidants are associated with risk of AD onset. Given that genetically determined differences in circulating exposures between individuals are lifelong and not affected by environmental traits, we hypothesized that AD risk would be lower among individuals with genetically increased circulating antioxidants if higher circulating antioxidant exposure does help to prevent AD.

## Methods

### Study design

A series of MR analyses were conducted using a 2-sample design (18, 19). MR entails the use of genetic variation to infer the effects of a modifiable (nongenetic) trait on outcomes of interest (17). The 2-sample approach requires the identification of genotypes that affect an exposure from ≥1 published genetic association studies for exposures of interest (the first samples) and then assessment of genotype-outcome associations for each identified genetic variant in secondary data sets, which have homogeneous characteristics (of similar genetic ancestry) to the first samples. The framework that underlies MR studies is depicted and described in **Supplemental Figure 1**, and detailed further elsewhere (17, 20).

### Genetic variants affecting circulating antioxidants

We considered identification of genetic variants as instrumental variables for the following 5 circulating antioxidants that are modified by dietary factors and which have been linked to AD risk in observational epidemiologic studies: *1*) α-tocopherol (a major molecule of the vitamin E family), *2*) ascorbic acid/ascorbate (vitamin C), *3*) β-carotene, *4*) retinol (vitamin A), and *5*) uric acid/urate. In searching for genetic instruments for the antioxidants, we prioritized consistent, replicated findings from genome-wide association studies (GWASs) for the identification of single-nucleotide polymorphisms (SNPs) that determine differences in the traits. We searched for GWASs of the circulating antioxidants of interest in the NHGRI-EBI GWAS catalog (http://www.ebi.ac.uk/gwas/) and in the wider literature via the PubMed search engine. We identified GWAS findings for circulating measures of each trait of interest, with the exception of ascorbate (21–24). However, for ascorbate, there are robust, replicated findings from a meta-analysis of SNPs at a locus with an established role in vitamin C metabolism, combining data on 15,087 participants in 5 cohort studies (25).

Next, we examined the suitability of the top SNPs from these studies as instruments for the exposures of interest. In MR, plausible instrumentation of exposures should ideally be based on 1 of the following 2 criteria: *1*) it is possible to use numerous variants from various loci that affect the exposure independently, which enables additional methods to test for violations of instrumental variable assumptions, and *2*) variants are located in or near single genes with established pathways linking the gene(s) specifically to effects on an exposure (26). With 30 independent SNPs used to instrument urate, this analysis fulfilled the first criterion. A genetic score for higher circulating urate is associated with the risk of gout (caused by hyperuricemia)—providing positive control evidence that these variants can be used to proxy urate exposure (21). Instrumenting of ascorbate, β-carotene, and retinol each fulfilled the second criterion. The SNP identified as an instrument for ascorbate is a missense variant in an exon of Solute Carrier Family 23 Member 1 (*SLC23A1*), which encodes sodium-dependent vitamin C transporter 1 (SVCT1), 1 of 2 cotransporters involved in the intestinal absorption and active transport of dietary ascorbate (25). SNPs instrumenting β-carotene are in beta-carotene oxygenase 1 (*BCO1*/alias *BCMO1*), which encodes the enzyme carotenoid 15,15′-monooxygenase, responsible for catalyzing the cleavage of carotenoids into retinal in the small intestine (22). Rare loss-of-function mutations in this gene can produce hypercarotenemia, because excess carotenoids are not removed from circulation (as required for hepatic storage in the form of vitamin A) (27). The SNP used to instrument retinol is located near retinol binding protein 4 (*RBP4*), encoding retinol-binding protein 4, the major carrier that facilitates the transport of retinol from liver stores to peripheral tissues. Deleterious mutations in *RBP4* produce nominal circulating retinol (28) and may lead to vitamin A–related disorders, such as retinitis pigmentosa (29). Although GWAS has identified 3 hits for circulating α-tocopherol (22, 23), these findings provided neither numerous instruments for the exposure nor the specificity of the SNPs as determinants of α-tocopherol only [the 3 loci play clear roles in lipid metabolism (22)]. We therefore chose not to proceed with MR analyses of α-tocopherol and AD risk.

**FIGURE 1:**
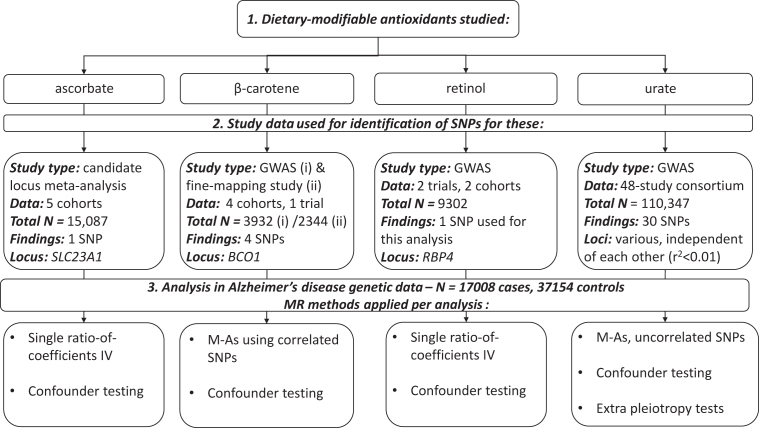
Flow chart summarizing the antioxidants studied, identification of genetic instruments, and data and MR methods used for analyses. *BCO1*,beta-carotene oxygenase 1; GWAS, genome-wide association study; IV, instrumental variable; M-A, meta-analysis; MR, Mendelian randomization; *RBP4*, retinol-binding protein 4; *SLC23A1*, solute carrier family 23 member 1; SNP, single-nucleotide polymorphism.

Genetic association study samples were largely from cohort studies representative of general populations (some samples were derived from RCTs), and all used data on participants of European ancestry only. **Figure 1** summarizes the variant selection, along with specific methods used for analyses of each antioxidant in relation to AD risk. A full list of SNP identifiers (rs numbers) and associated information for variants used in the analyses are shown in **Supplemental Table 1**.

### The AD case-control sample

The sample for examining genotype-outcome associations consisted of 17,008 late-onset AD cases and 37,154 controls of European ancestry included in the stage 1 GWAS meta-analysis conducted by the International Genomics of Alzheimer's Project (IGAP) (30). IGAP has published summary statistics of genotype-AD associations for 7,055,881 SNPs online (http://web.pasteur-lille.fr/en/recherche/u744/igap/igap_download.php). Cases within the consortium's cohorts had mean ages of onset ranging from 68.5 to 82.3 y, and $\sim \! 60 \%$ were women. More details on the stage 1 studies, participants, genotype data, AD diagnostic criteria, and statistical models are described in the published GWAS (30), and constituent studies are listed in **Supplemental Table 2**. The analyses included adjustment for principal components to control for population stratification, which could bias GWASs (and thus MR results as well) if not accounted for.

### Sample overlap

We attempted to quantify the degree of overlap between participants included in the GWAS of antioxidants and the study by the AD consortium, which could bias 2-sample MR results if substantial (31). The online supporting material includes full commentary (**Supplemental Methods**) and details of cohort numbers (Supplemental Table 2 and**Supplemental Tables 3–5**). The risk of bias from sample overlap in all analyses appeared to be low.

### Main models

For each SNP associated with circulating antioxidants, we extracted β coefficients and SEs for the SNPs’ effects on antioxidant concentrations from published genetic association studies of the traits and also the corresponding β coefficients and SEs for the SNP associations with AD risk from IGAP data (reported as differences in log-odds). These statistics were harmonized to ensure that they corresponded to the same forward strand allele for each SNP, all of which increase antioxidant concentrations.

MR estimates of the magnitudes to which long-term variation in antioxidants might affect AD risk (the primary outcome) were produced using the ratio of coefficients method (19). Wald estimators were calculated for each SNP by dividing the estimated β coefficient for its association with AD risk by the β coefficient for its association with antioxidant. SEs for each estimator were calculated by the delta method (32).

For urate, the estimates for all variants were combined in a fixed-effects meta-analysis using inverse-variance weighting (IVW) models (i.e., combining 30 individual SNP estimates). The overall IVW meta-analysis results provide more precision for effect estimation than individual SNP-AD associations alone. The genotypes of individual SNPs used in combinations for the 3 traits were not correlated [i.e., not in linkage disequilibrium (LD)] and so each of the several AD effect estimates were independent of one another. For β-carotene, we combined estimators from 4 variants at a single locus (the *BMCO1* gene) that were assessed in a fine-mapping association study of β-carotene with genetic variation in this gene (33), expanding on the findings from a previous GWAS of β-carotene (22). These 4 variants were modestly correlated due to LD (all pairwise Pearson's *r* ≤ ±0.32; *R*^2^ < 0.2) but still explain more variation in circulating β-carotene when combined than the single strongest GWAS hit at the locus does alone (33). The use of multiple correlated variants together therefore increases the statistical power for estimating the effect of β-carotene variation on AD risk, but would also bias the precision of an IVW estimate without appropriate weighting for the correlated variants, because the constituent individual estimates are assumed to be independent (34). Instead, we used an extension of the IVW method for the meta-analysis of β-carotene estimates, which included adjustment for a matrix of correlations between the SNPs (shown in **Supplemental Table 6**) (35). The matrix was derived from SNP correlations in reference data on participants of European ancestry in the 1000 Genomes project, phase 3 (36). Models for ascorbate and retinol used single Wald estimators for the sole SNPs that instrument these antioxidants specifically, and thus meta-analysis models were not conducted for these traits.

All results were reformatted by exponentiation to be expressed as ORs and 95% CIs for AD per long-term genetically predicted higher exposure to the circulating antioxidants. These results correspond to increased exposure in original units of measure for urate (milligrams per deciliter) and ascorbate (micromoles per liter). The GWASs of β-carotene and retinol were conducted on log_n_-transformed values; for ease of interpretation, we reformatted the ratio of geometric means and SEs for SNP associations with the antioxidants as relative percentage differences (37). Ensuing Wald estimators for these 3 traits were then scaled to be expressing AD risk as ORs with CIs according to 10% relative increases in concentrations of β-carotene and retinol.

### Antioxidant associations with AD risk factors

As shown in Supplemental Figure 1, key model assumptions in MR can be violated by horizontal pleiotropy, where variants affect outcome risk via combinations of potential confounders and alternate pathway(s), independently of their effects on the exposures of interest (20). To test whether this could bias the main findings, we examined whether the SNPs used as instruments may also be determinants of several other major risk factors for (and potential causes of) AD. We addressed several traits for which published genome-wide summary statistics are also available in open access: years of education attained, likelihood of smoking initiation, adiposity (measured by BMI), and cardiometabolic traits (triglycerides, LDL cholesterol, fasting glucose, and insulin). The GWAS data sets used for these analyses are described in **Supplemental Table 7**. All participants were of European ancestry, and sample sizes ranged from 21,544 to 322,154. For MR estimates of effects of antioxidants on these traits, we performed the same primary MR analyses as were conducted for main AD analyses, that is, using IVW methods for urate and β-carotene analyses and producing single Wald estimators for ascorbate and retinol.

### Further pleiotropy tests for urate analysis

In the urate analysis, where we had multiple independent SNPs to instrument the exposure, we performed a series of additional checks for evidence of bias on MR effect estimates dues to horizontal pleiotropy. In brief, these involved the use of meta-analysis heterogeneity statistics, alternate MR methods (weighted median, weighted mode, and MR-Egger analyses), funnel plotting, and a sensitivity analysis (26, 38–41). Full details on these analyses are described in the Supplemental Methods. These pleiotropy-testing methods were not applicable for the ascorbate, β-carotene, and retinol analyses due to the small number of SNPs being used to instrument these traits.

### Additional sensitivity analyses

Where IVW models were conducted (for urate and β-carotene), we repeated analyses using a likelihood-based approach, which encompasses the uncertainty in SNP-antioxidant exposures more accurately than the simple weighting applied in IVW (42). The likelihood model for the β-carotene–AD analysis included adjustment for SNP correlations, as described for the corresponding IVW model.

### Power calculations and tests of "weak instrument" bias

To examine whether we had a sufficient sample size to undertake the MR analyses, we conducted power calculations using a published calculator (43). This estimated the power for analyses to detect minimum ORs for AD risk per SD difference in antioxidant concentrations. The calculations used the study-level average *R*^2^ statistics for variance explained in each antioxidant by the combination of SNPs determining trait variation (ranging from 0.5% to 7.0%), along with the sample size (*n* = 54,162) and proportion of cases (0.314) in the stage 1 IGAP sample.

To assess whether instrumental variable models could be biased by the use of "weak instruments" (where variants may not be robustly confirmed determinants of antioxidant variation), we examined any reported *F* statistics from genetic association analyses of the SNPs in use and corresponding antioxidants and estimated the anticipated *F* values from reported *R*^2^ values and sample sizes used in these studies where these were not found directly reported in the relevant publications.

### Software

Analyses were conducted in R software version 3.2.2, with the use of packages MR Base and MendelianRandomization (44, 45). Plots were produced in Stata version 15.1 (StataCorp LLC) using the package mrrobust (46).

### Ethics

This research involved only the reuse of existing published results and study-level summarized data and therefore did not require separate ethical approval. All genetic association studies of circulating antioxidants had obtained relevant ethical approval and informed consent from study participants (21–25, 33). Written informed consent was obtained from study participants in IGAP or, for those with substantial cognitive impairment, from a caregiver, legal guardian, or other proxy instead. IGAP study protocols were reviewed by the local or institutional ethics review boards of the consortium's members (30). All accorded with the Declaration of Helsinki.

## Results

**FIGURE 2:**
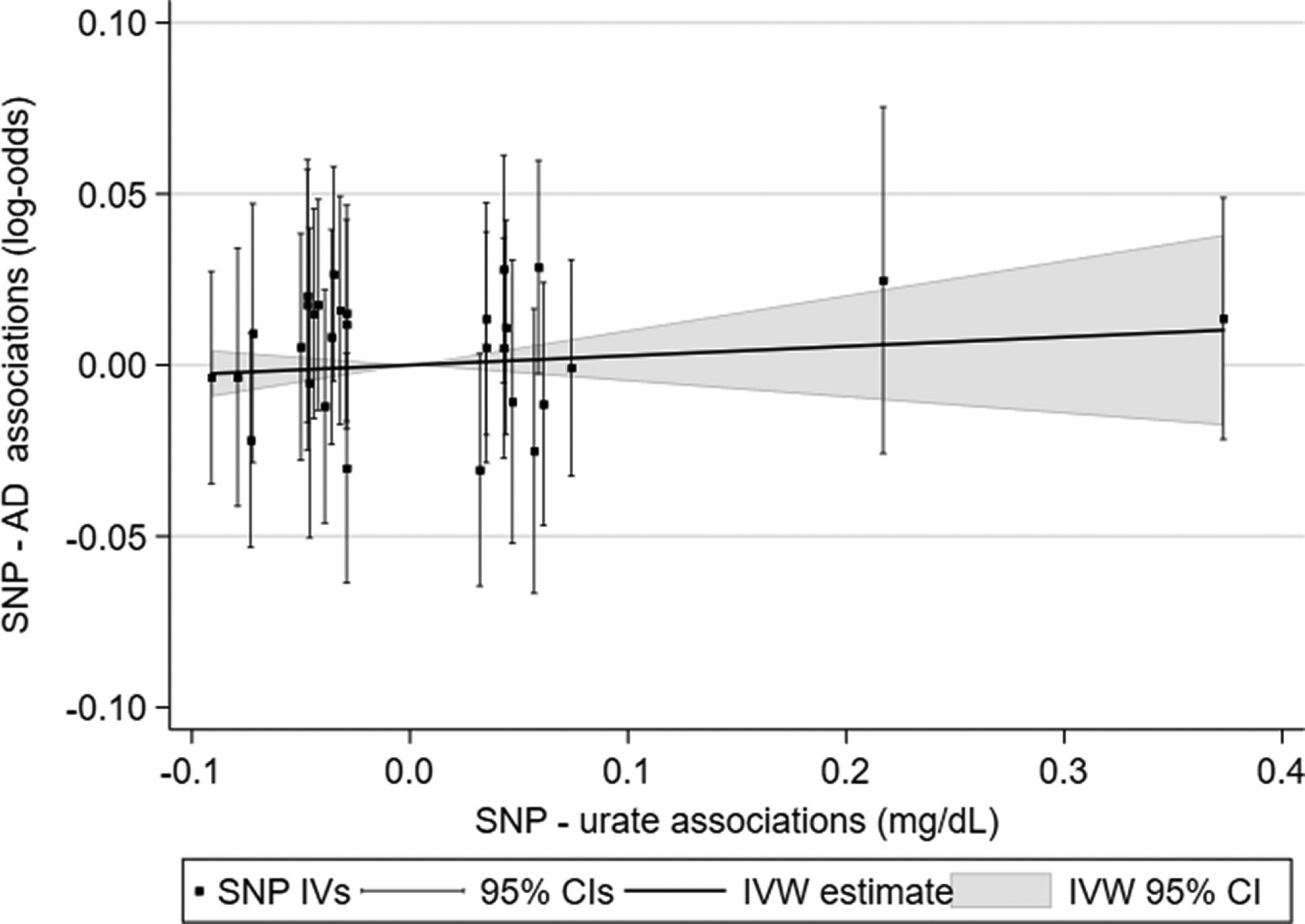
Scatterplot showing estimates of AD risk according to urate exposure. All 30 individual IV estimates of urate-AD associations are plotted according to their effects on urate (*x* axis) and on AD risk (*y*axis). The IVW meta-analysis result (corresponding to the ORs and CIs listed in Table 1) is plotted through individual estimates, with no strong deviation from the null indicating a lack of effect of urate exposure on AD risk. AD, Alzheimer disease; IV, inverse variance; IVW, inverse-variance weighted; SNP, single-nucleotide polymorphism.

In primary results (**Table 1**) there were no apparent differences in AD risk according to genetically predicted increases in exposure to circulating ascorbate, retinol, or urate. Higher predicted exposure to circulating β-carotene was associated with marginally elevated AD risk—although the OR was similar to results for other antioxidants, it was estimated with more precision. **Figure 2** shows a scatterplot of individual urate results, along with the meta-analysis IVW estimate; the lack of trend indicates a null finding.

**TABLE 1:** Associations of long-term, genetically increased circulating antioxidants with AD risk from MR analyses^1^

Trait	Number of SNPs determining trait variation	Approximate total variance in trait explained by SNPs,^2^ %	MR estimate for AD risk, OR (95% CI)^3^
Ascorbate	1	0.9	1.00 (0.99, 1.02)
β-Carotene	4	6.1	1.02 (1.00, 1.03)
Retinol	1	0.5	1.05 (0.94, 1.18)
Urate	30	7.0	1.03 (0.96, 1.10)

1 *n* = 17,008 cases and 37,154 controls. AD, Alzheimer disease; MR, Mendelian randomization; SNP, single-nucleotide polymorphism.

^2^Variances are *R*^2^ values (reformatted as percentages) reported in relevant genetic association studies (21, 24, 33, 47).

3 Results are based on the fixed-effects inverse-variance weighted method for meta-analyzing individual SNP results (urate and β-carotene) or single SNP Wald estimators (ascorbate and retinol). ORs (95% CIs) show risk of AD per long-term unit increase in exposure to circulating ascorbate (μmol/L) and urate (mg/dL) and per long-term 10% higher exposure to circulating β-carotene and retinol.

**FIGURE 3:**
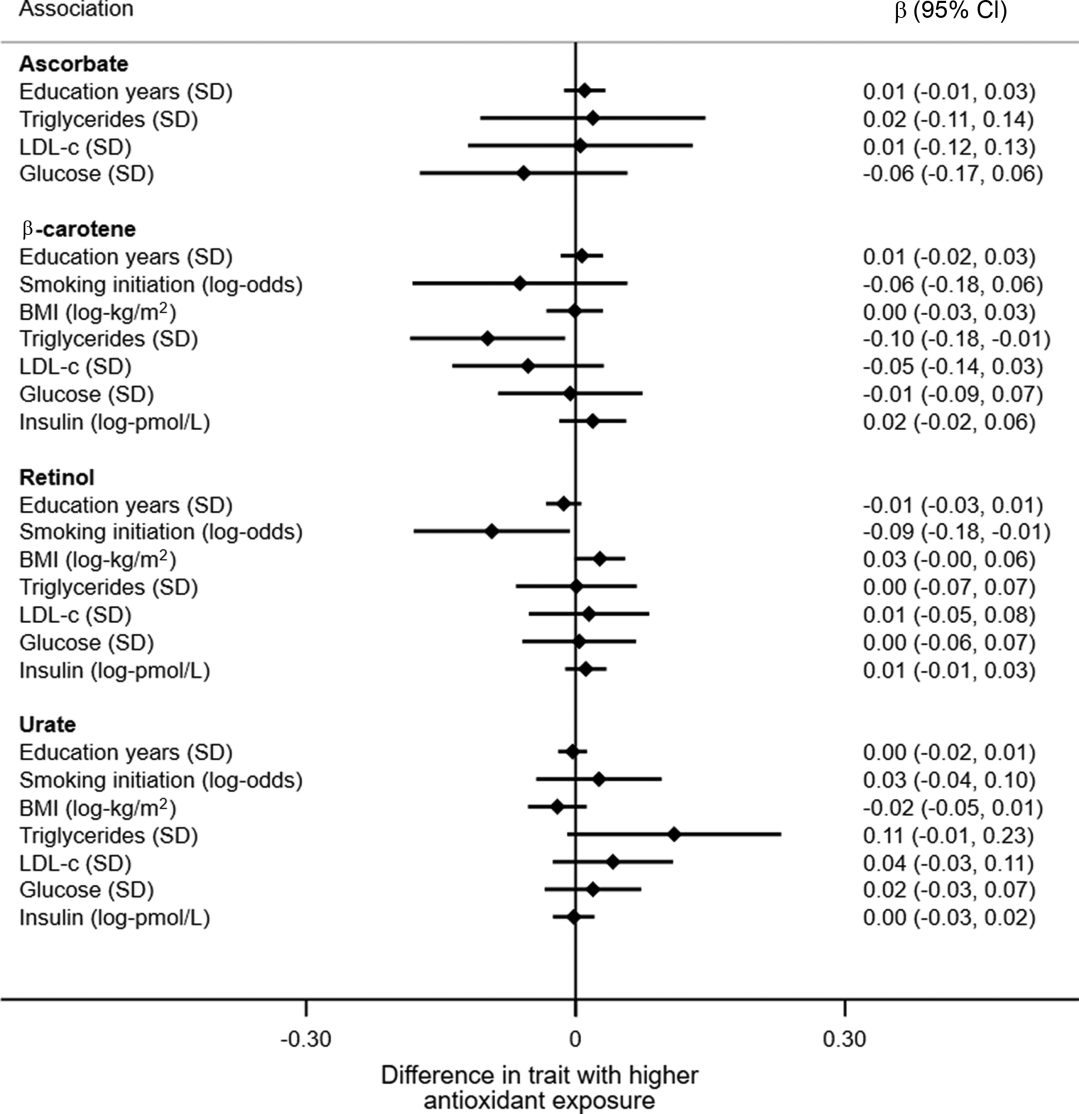
MR results for antioxidant exposure and traits that may also determine AD risk. Results are based on the IVW method for meta-analyzing individual SNP results (urate and β-carotene) or single SNP Wald estimators (ascorbate and retinol). β Coefficients and 95% CIs show trait unit differences (in parentheses) per 10-µmol/L higher circulating ascorbate, mg/dL higher urate, log-µmol/L higher β-carotene, and 10% higher retinol. AD, Alzheimer disease; IVW, inverse-variance weighted; LDL-c, LDL cholesterol; MR, Mendelian randomization; SNP, single nucleotide polymorphism.

**Figure 3** shows MR estimates for effects of antioxidant exposure on AD risk factors. Overall, there was little evidence for associations of genetically predicted differences in circulating antioxidants with lifestyle and cardiometabolic traits. The most prominent exceptions were inverse associations of β-carotene with triglycerides, and retinol with smoking initiation, and possible associations of retinol with higher BMI and urate with higher triglycerides (however, no association would survive Bonferroni correction for multiple testing).

**Supplemental Table 8** shows results of MR estimates for the effect of exposure to urate on AD risk produced with 4 models that differ from IVW—namely, a maximum likelihood alternative and weighted median, weighted mode, and MR-Egger methods. The results were largely consistent with the primary IVW model. There was no suggestion of overall directional (nonneutral) bias from pleiotropy in estimates from the MR-Egger intercept test (*P* = 0.22) or that the MR-Egger estimate of effect would be biased toward the null by measurement error in urate instrumentation (*I*^2^_gx_ = 99.4%) (48). Heterogeneity statistics indicated that there was limited inconsistency between individual SNP estimates within meta-analyses (IVW model Q test *P* value = 0.48), also indicative of no pleiotropic bias; a forest plot depicts this consistency (**Supplemental Figure 2**).

Additional steps taken to further assess whether pleiotropy had influenced the results for the effect of urate on AD risk also indicated no substantial bias in the findings. The funnel plot for urate results showed no asymmetry, with results consistently spread around the null irrespective of the size of the effect on urate (**Supplemental Figure 3**). In the sensitivity analyses using a subset of 14 SNPs as instruments for urate, results from all MR models were consistent with those using the 30 SNPs but with slightly less precision. For example, the OR for AD risk from the fixed-effects IVW estimate in the subset analysis was 1.03 (95% CI: 0.94, 1.12).

Illustrative power calculations suggested that the IGAP sample size and genetic instrumenting of β-carotene and urate should allow for identification of small associations with AD risk in MR models (**Supplemental Table 9**). In contrast, the lower variances in circulating ascorbate and retinol that are instrumented by SNPs in these analyses implies that variation in exposure to these traits would need to have moderate to large effects on AD risk for identification of genetically predicted associations to be probable in these MR models.

An evaluation of observed or estimated *F* statistics from genetic association analyses of variants and the antioxidants suggested no evidence of weak instrument bias in analyses. All *F* values were >10, the threshold under which weak instrument bias may be expected (42). A previous MR study using the variant rs33972313 as an instrument for circulating vitamin C exposure reported an *F* value of 30 for the SNP-ascorbate association, estimated from a sample size of 3512, and an *R*^2^ of 0.009 for variance in ascorbate explained by the SNP (47). *F* values estimated from sample sizes and *R*^2^ values of other analyses were $\sim \! 152$ for the combined β-carotene instruments (from *n* = 2344; *R*^2^ = 0.061) (33), $\sim \! 47$ for the single retinol SNP (*n* = 9302; *R*^2 ^= 0.005) (24), and $\sim \! 8305$ for the combined urate instruments (*n* = 110,347; *R*^2^ = 0.07) (21).

## Discussion

The findings of these MR analyses suggest that increasing individuals’ long-term exposure to circulating ascorbate, β-carotene, retinol, and urate would not mitigate their risk of developing AD. All estimates of effects of antioxidant exposure on AD risk were close to null, with ORs for retinol, β-carotene, and urate >1, implying that higher exposure confers slightly more risk of AD (if any difference at all), rather than neuroprotection.

These MR results contrast with findings from several sources of conventional epidemiologic evidence (summarized in **Table 2** for ease of reference). In meta-analyses of AD case-control studies with circulating micronutrient measures, combined estimates suggested AD cases have lower concentrations of α-tocopherol, ascorbate, and retinol than controls (β-carotene studies were not included) (4). Prospective studies addressing associations of these exposures with later AD incidence to date have relied on baseline dietary intake measures, rather than assay data (10, 49–54). A meta-analysis reported associations of lower intake of ascorbate, β-carotene, and vitamin E with later risk of AD onset (retinol studies were not addressed) (8). Elevated exposure to urate—regarded as a potent endogenous antioxidant, influenced by dietary intake of purines (55, 56)—has also been proposed as a candidate for AD prevention (57). National register–based studies have observed AD and dementia incidences to be lower than expected among aging individuals with a history of gout, a condition caused by hyperuricemia (6, 58). Moreover, in meta-analyses of case-control and prospective studies, lower circulating urate was also associated with higher risk of both AD (combining 24 studies) and all-cause dementia (combining 31 studies) (59, 60). However, a recent umbrella review highlighted a lack of credibility of these meta-analysis results (61).

**TABLE 2:** Summary of meta-analyses and other large-scale observational data on associations of 4 major antioxidants and AD risk^1^

Antioxidant	Study type (reference)	Exposure measurement	Sample size, cases; noncases^2^	Main findings: association estimate (95% CI)
Ascorbate	Meta-analysis of case-control studies (4)	Circulating concentrations	223; 211	Age-adjusted mean difference in cases: –14.2 μmol/L (–22.2, –6.3 μmol/L)
	Meta-analysis of prospective studies (8)	Estimated dietary intake of vitamin C	1043; 13,468	Relative risk for highest intake group: 0.83 (0.72, 0.94)
β-Carotene	Meta-analysis of prospective studies (8)	Estimated dietary intake of carotenoids	801; 9445	Relative risk for highest intake group: 0.88 (0.73, 1.02)
Retinol	Meta-analysis of case-control studies (4)	Circulating concentrations	310; 674	Age-adjusted mean difference in cases: –0.4 μmol/L (–0.6, –0.2 μmol/L)
Urate	Meta-analysis of case-control studies (59)	Circulating concentrations	1128; 2498	Weighted mean difference: –0.77 mg/dL (–1.18, –0.36 mg/dL)
	Meta-analysis of prospective studies (59)	Circulating concentrations	661; 6666^3^	Risk ratio in group with highest concentrations: 0.66 (0.52, 0.85)
	Register-nested prospective study (6)	History of gout (indicative of high urate exposure)	2251; 295,778	Adjusted AD HR in gout cases (high urate exposure group): 0.76 (0.66, 0.87)

1 AD, Alzheimer disease.

^2^Numbers reflect case-control samples, or for prospective studies, incident cases and healthy individuals at end of follow-up.

3 These numbers reflect minimum incident dementia or AD cases (this is not explicitly stated in 1 of 3 studies that was meta-analyzed) from a total sample of 7327 listed in the meta-analysis.

Systematic biases in past observational studies could explain the disparities between previous findings and these MR results. Studies of dietary components as health exposures may be particularly prone to residual confounding, because dietary factors are highly correlated with one another (62) and with a multitude of other lifestyle and socioeconomic traits. Hence, antioxidant status may closely proxy wider risk factors for AD, and particularly overall dietary patterns, which could have complex effects on disease risk that are not produced by sole micronutrient measures individually. Case-control studies often cite associations of antioxidants with AD status as evidence of the potential for antioxidant modulation to prevent AD, but these findings may have arisen from reverse causation. For instance, AD patients may have altered intake, uptake, or utilization of antioxidants; that is, lower circulating antioxidant concentrations in AD cases may reflect disease-driven physiologic differences or malnutrition (4, 63–65). In contrast, MR studies can circumvent bias from confounding and reverse causation. There could be other important violations to MR assumptions (discussed below), but if these findings are reliable, they would suggest that previous observational studies may have overstated the role of circulating concentrations of antioxidants in AD develop-ment.

The major strength of this study was the use of MR analyses, which differ substantially from prior studies and add to the evidence base for causal inference regarding these questions. The use of the 2-sample design and the very large volume of case-control data allowed analyses with sufficient power to detect even small to modest effects for β-carotene and urate (although with less power for ascorbate and retinol). Various methodologies were utilized to examine for model violations (chiefly from pleiotropy) in different ways, increasing the robustness of most results.

Two-sample MR models have several general limitations, including assumptions of linear associations, lifelong (not time-sensitive) effect estimations, and the possibilities of inference being biased by genetic phenomena such as canalization and confounding by LD between variants or sample substructures, i.e., population stratification (controlled for by principal components in these AD models)—these are discussed in detail elsewhere (17, 31, 66). However, there are also specific constraints on the current evidence due to the SNPs used to instrument antioxidants. First, the use of variants at single loci to instrument ascorbate, β-carotene, and retinol precluded several sensitivity analyses to check for bias in these 3 results due to pleiotropy (as were conducted for urate). Second, the use of single SNPs to instrument ascorbate and retinol also limited the power of these analyses, even with the very large AD case-control sample available. Larger GWASs that confirm many independent SNPs as instruments for these antioxidants would improve further MR studies by both increasing power and allowing for more nuanced testing of bias due to pleiotropy. Third, although β-carotene was instrumented more strongly than ascorbate and retinol, using multiple variants at the *BCO1* locus, this gene appears to have antagonistic effects on different carotenoid concentrations; that is, alleles increasing β-carotene and other major carotenoids also lower lycopene and lutein (22). Rather than predicting β-carotene variation solely, these results should therefore be regarded as instrumenting more complex changes in carotenoid concentrations affected by this gene region simultaneously. However, any inference based on the use of these variants may still largely mimic any effects of β-carotene supplementation, were major carotenoid supplementation to similarly displace minor carotenoids from circulation (22, 67).

In conclusion, this evidence casts doubt on the role of several circulating antioxidants in AD prevention and suggests caution toward planning RCTs to test the effect of related nutritional supplements or urate-elevating therapeutics on AD risk. Future MR studies could aim to expand on these findings with larger replication samples of genetic data on AD cases and controls, ideally using a higher number of variants to instrument each exposure, if more are identified through increasingly large GWASs of circulating antioxidants (which might also permit robust analyses for vitamin E). Where genetic data become available for AD patients with disease progression measures, future MR studies could also help to evaluate whether circulating antioxidant modifications may provide disease-modifying treatments, even if not useful traits to consider for disease prevention (68).

## Supplementary Material

nqy225_Supplemental_File

## Notes

Supported by a European Union Horizon 2020 Research and Innovation Program grant (agreement 634821), the Swedish Council for Working Life and Social Research (grant 2013-2292), and the Swedish Research Council (grants 521-2013-8689, 2015-03255, 2015-06796, and 2015-02907). For the International Genomics of Alzheimer's Project, the i-Select chips were funded by the French National Foundation on Alzheimer disease and related disorders. The European Alzheimer's Disease Initiative was supported by the LABEX (Laboratory of Excellence Program Investment for the Future) DISTALZ grant, Inserm, Institut Pasteur de Lille, Université de Lille 2, and the Lille University Hospital. The Genetic and Environmental Risk in Alzheimer's Disease was supported by the Medical Research Council (grant 503480), Alzheimer's Research UK (grant 503176), the Wellcome Trust (grant 082604/2/07/Z) and the German Federal Ministry of Education and Research (BMBF): Competence Network Dementia grants 01GI0102, 01GI0711, and 01GI0420. The Cohorts for Heart and Aging Research in Genomic Epidemiology was partly supported by the NIH/National Institute on Aging (NIA) grant R01 AG033193 and the NIA AG081220 and AGES contract N01-AG-12100, the National Heart, Lung, and Blood Institute grant R01 HL105756, the Icelandic Heart Association, and the Erasmus Medical Center and Erasmus University. The Alzheimer's Disease Genetics Consortium was supported by NIH/NIA grants U01 AG032984, U24 AG021886, and U01 AG016976 and Alzheimer's Association grant ADGC-10-196728. The funders had no role in the study design, data collection and analysis, decision to publish, or preparation of this manuscript.

Supplemental Figures 1–3, Supplemental Methods, and Supplemental Tables 1–9 are available from the “Supplementary data” link in the online posting of the article and from the same link in the online table of contents at https://academic.oup.com/ajcn/.

Abbreviations used: AD, Alzheimer disease; GWAS, genome-wide association study; IGAP, International Genomics of Alzheimer's Project; IVW, inverse-variance weighted; LD, linkage disequilibrium; MR, Mendelian randomization; RCT, randomized controlled trial; SNP, single-nucleotide polymorphism.
