# Supplementary material for: Circulating antioxidants and Alzheimer disease prevention: a Mendelian randomization study
Source: Am J Clin Nutr. 2018 Dec 29;109(1):90–8. doi: 10.1093/ajcn/nqy225 (PMC6358036; doi:10.1093/ajcn/nqy225)
Supplement: nqy225_Supplemental_File [file nqy225_supplemental_file.docx]

The role of circulating antioxidants in Alzheimer’s disease prevention: a Mendelian randomization study

Dylan M. Williams, Sara Hägg, Nancy L. Pedersen.

**Supplemental methods**

*Sample overlap*

Supplemental tables 2 to 4 show cohorts and numbers of participants in each genetic association study used in this analysis, and supplemental table 5 shows the approximate overlap of samples between GWAS of urate and AD. The precise degree of overlap could not be determined, but of 143,160 participants whose data were in the urate GWAS, the maximum number whose data could have also been in the AD GWAS was approximately 8795 (6% of the urate study’s total sample). There was no apparent overlap between GWAS of other antioxidants and the IGAP sample, assuming that participants were not members of several cohorts included – unlikely in most instances, given the geographic spread of the cohort studies. Risk of bias from sample overlap in each exposure and outcome analysis is therefore likely to be low.

*Additional analyses to test and account for pleiotropy in urate models*

For the urate meta-analysis model, we plotted single SNP results separately to examine for outliers and used the models’ heterogeneity statistics -- Cochran’s Q and I^2^ values-- to examine for differences in estimates are present: if true underlying effects of urate on disease risk are present, individual estimates should cluster consistently around the true magnitudes (1).

Second, we repeated urate analyses with several methods that are designed to account for some degree of horizontal pleiotropy with weaker model assumptions for testing causality: weighted median and modal-based estimators, and MR-Egger regression (2-4). These have subtly different sets of instrumental variable assumptions to IVW models, and different discriminative properties for estimating magnitudes of effects of exposures on outcome when some proportion of horizontal pleiotropic effects amongst variants exist (for a comparison of the differences, refer to (3, 5)). The test of the intercept in MR-Egger models differing from zero can give an indication of bias from an overall (non-neutral) effect of pleiotropy, so these test statistics were also calculated (4). Median, mode and MR-Egger methods require a minimum of three independent instruments for exposures to conduct, so the additional models were not applied in the ascorbate, β-carotene and retinol analyses.

We also produced a funnel plot of MR estimates from each SNP individually. This shows the variation in individual estimates of AD risk according to the precision of each instrument for predicting urate variation: asymmetry in a plot indicates an overall imbalance in pleiotropic effects between the variants, which could bias the summary estimate away from the null in either direction (i.e. directional pleiotropy) (5). Funnel plots using the small number of variants as instruments in other analyses (N≤4) are not informative, so only urate results were plotted.

Some of the SNPs used in the urate analysis were associated with a number of physiological traits in a previous MR study of urate in relation to cardiometabolic diseases, with some potentially confounding any antioxidant-AD association, and many of the traits could also be factors on pathways affecting AD risk independently of urate variation (by horizontal pleiotropy) (6). We therefore conducted a sensitivity analysis, repeating all urate models using 14 of the SNPs that were not implicated in pleiotropic pathways in the previous MR study (6).

**Supplemental table 1: Information on SNPs used in each set of analyses**

|  | **SNP ID** | **Chr** | **Position ^1^** | **Nearest gene** | **Coded allele** | **Alt. allele** | **Coded allele freq. ^2^** | **AD risk per coded allele copy; OR (95% CI)** |
| --- | --- | --- | --- | --- | --- | --- | --- | --- |
|  |  |  |  |  |  |  |  |  |
| *Ascorbate* |  |  |  |  |  |  |  |  |
| 1 | rs33972313 | 5 | 139379813 | *SLC23A1* | T | C | 0.04 | 1.02 (0.93, 1.12) |
|  |  |  |  |  |  |  |  |  |
| *β-carotene* |  |  |  |  |  |  |  |  |
| 1 | rs12934922 | 16 | 81268089 | *BCO1* | T | A | 0.44 | 1.03 (1.00, 1.07) |
| 2 | rs4448930 | 16 | 81297397 | *BCO1* | C | G | 0.15 | 1.01 (0.96, 1.06) |
| 3 | rs4889286 | 16 | 81223108 | *BCO1* | T | C | 0.50 | 1.02 (0.99, 1.06) |
| 4 | rs7501331 | 16 | 81280891 | *BCO1* | T | C | 0.24 | 1.00 (0.95, 1.03) |
|  |  |  |  |  |  |  |  |  |
| *Retinol* |  |  |  |  |  |  |  |  |
| 1 | rs10882272 | 10 | 93588425 | *FFAR4/RBP4* | C | T | 0.35 | 0.98 (0.95, 1.02) |
|  |  |  |  |  |  |  |  |  |
| *Urate* |  |  |  |  |  |  |  |  |
| 1 | rs10480300 | 7 | 151708919 | *PRKAG2* | T | C | 0.28 | 1.01 (0.98, 1.05) |
| 2 | rs10821905 | 10 | 50886333 | *A1CF* | A | G | 0.18 | 0.98 (0.94, 1.02) |
| 3 | rs11264341 | 1 | 155179017 | *TRIM46* | T | C | 0.43 | 1.00 (0.97, 1.04) |
| 4 | rs1165151 | 6 | 25821388 | *SLC17A1* | T | G | 0.47 | 1.00 (0.97, 1.03) |
| 5 | rs1171614 | 10 | 59709780 | *SLC16A9* | T | C | 0.22 | 1.00 (0.96, 1.04) |
| 6 | rs1178977 | 7 | 73442719 | *MLXIPL* | G | A | 0.81 | 1.01 (0.97, 1.05) |
| 7 | rs12498742 | 4 | 9942428 | *SLC2A9* | G | A | 0.77 | 0.99 (0.95, 1.02) |
| 8 | rs1260326 | 2 | 27508073 | *GCKR* | T | C | 0.41 | 1.00 (0.97, 1.03) |
| 9 | rs1394125 | 15 | 75866642 | *UBE2Q2* | A | G | 0.34 | 1.03 (0.99, 1.06) |
| 10 | rs1471633 | 1 | 145711327 | *PDZK1* | A | C | 0.46 | 1.03 (1.00, 1.06) |
| 11 | rs17050272 | 2 | 120548864 | *INHBB* | A | G | 0.43 | 1.01 (0.97, 1.04) |
| 12 | rs17632159 | 5 | 73135655 | *TMEM171* | C | G | 0.31 | 0.99 (0.95, 1.02) |
| 13 | rs17786744 | 8 | 23919493 | *STC1* | G | A | 0.58 | 0.99 (0.95, 1.02) |
| 14 | rs2078267 | 11 | 64566642 | *SLC22A11* | C | T | 0.51 | 1.02 (0.99, 1.05) |
| 15 | rs2231142 | 4 | 88131171 | *ABCG2* | T | G | 0.11 | 1.03 (0.97, 1.08) |
| 16 | rs2307394 | 2 | 147958859 | *ACVR2A* | C | T | 0.68 | 1.03 (1.00, 1.07) |
| 17 | rs2941484 | 8 | 75566533 | *HNF4G* | T | C | 0.44 | 1.01 (0.98, 1.04) |
| 18 | rs3741414 | 12 | 57450266 | *INHBC* | T | C | 0.24 | 1.01 (0.97, 1.04) |
| 19 | rs478607 | 11 | 64710591 | *NRXN2* | G | A | 0.84 | 0.98 (0.94, 1.03) |
| 20 | rs642803 | 11 | 65793149 | *OVOL1* | T | C | 0.46 | 1.01 (0.98, 1.04) |
| 21 | rs653178 | 12 | 111569952 | *ATXN2* | C | T | 0.51 | 0.97 (0.94, 1.00) |
| 22 | rs6598541 | 15 | 98727906 | *IGF1R* | A | G | 0.36 | 1.01 (0.97, 1.04) |
| 23 | rs675209 | 6 | 7101851 | *RREB1* | T | C | 0.27 | 0.99 (0.95, 1.02) |
| 24 | rs6770152 | 3 | 53066198 | *MUSTN1* | G | T | 0.58 | 0.99 (0.96, 1.02) |
| 25 | rs7188445 | 16 | 79701090 | *MAF* | A | G | 0.33 | 1.02 (0.98, 1.05) |
| 26 | rs7193778 | 16 | 69529987 | *NFAT5* | C | T | 0.86 | 1.01 (0.96, 1.05) |
| 27 | rs7224610 | 17 | 55287427 | *HLF* | C | A | 0.58 | 0.98 (0.95, 1.01) |
| 28 | rs729761 | 6 | 43836834 | *VEGFA* | T | G | 0.3 | 1.02 (0.98, 1.06) |
| 29 | rs7953704 | 12 | 122141445 | *B3GNT4* | A | G | 0.47 | 1.01 (0.98, 1.04) |
| 30 | rs7976059 | 12 | 51857488 | *ACVR1B* | T | G | 0.35 | 0.97 (0.94, 1.00) |

Chr: chromosome; SNP: single nucleotide polymorphism.

^1^ Positions based on reference data from NCBI Human Genome Build 38, patch 7.

^2^ Coded allele frequencies are according to those in the GWAS samples of studies of each circulating antioxidant where the SNP associations were identified.

**Supplemental table 2: Samples of AD cases and controls included in the stage 1 analysis of the IGAP GWAS (adapted from supplementary table 1 in (7))**

| **Cohort** | **N of cases included** | **Presumed N of controls ^1^** |
| --- | --- | --- |
| ACT | 566 | 1696 |
| ADC1 | 1411 | 515 |
| ADC2 | 737 | 160 |
| ADC3 | 364 | 570 |
| ADNI | 268 | 173 |
| AGES-RS | 78 | 2694 |
| CHS ^1^ | 421 | 1834 |
| EADI | 2243 | 6017 |
| FHS ^1^ | 183 | 3151 |
| GERAD (inc. Kora F4) | 3177 | 7277 |
| GSK | 669 | 713 |
| LOAD | 1811 | 1572 |
| MAYO | 728 | 1173 |
| MIRAGE | 509 | 742 |
| OHSU | 131 | 153 |
| ROSMAP | 291 | 776 |
| RS ^1^ | 633 | 5238 |
| TGEN2 | 129 | 493 |
| UMVUMSS | 1070 | 1128 |
| UPITT | 1271 | 841 |
| WASHU | 318 | 187 |
| Total (actual Ns cited) ^1^ | 17008 | 37154 |

For all cohort abbreviations, please refer to original study publications.

^1^ Precise numbers of controls included were not inferable from stated sample sizes in the IGAP GWAS article, due to disparities in the control numbers from prospective studies (CHS, FHS, RS) not being clarified.(7) The authors state that the stage 1 IGAP sample included 37154 controls in total.

**Supplemental table 3: Cohorts and participant numbers (for samples of Europeans only) included in the GWAS of circulating urate (8)**

| **Cohorts** | **N** |
| --- | --- |
| *Discovery samples* |  |
| AGES-RS ^1^ | 3219 |
| Amish | 1139 |
| ARIC | 9049 |
| ASPS | 845 |
| AUSTWIN | 11520 |
| BLSA | 521 |
| BRIGHT | 1743 |
| CARDIA | 1713 |
| CHS ^1^ | 3252 |
| CoLaus | 5409 |
| CROATIA-KORCULA | 895 |
| CROATIA-SPLIT | 490 |
| CROATIA-VIS | 912 |
| DESIR | 716 |
| EPIC-Norfolk cohort | 1835 |
| ERF | 889 |
| Estonian Biobank | 931 |
| Family Heart Study (FamHS) | 3837 |
| FHS ^1^ | 7699 |
| Health 2000 | 2069 |
| InCHIANTI | 1205 |
| INCIPE | 940 |
| INGI-Carlantino | 432 |
| INGI-CILENTO | 859 |
| INGI-FVG | 1018 |
| INGI-Val Borbera | 1658 |
| KORA F3 | 1643 |
| KORA F4 ^1^ | 1814 |
| LBC1936 | 769 |
| LifeLines | 3343 |
| LOLIPOP_EW_A | 587 |
| LOLIPOP_EW_P | 650 |
| LOLIPOP_EW610 | 924 |
| LURIC | 963 |
| MICROS | 1236 |
| NESDA | 1731 |
| NSPHS | 655 |
| ORCADES | 888 |
| PREVEND | 3785 |
| PROCARDIS | 3742 |
| RS-I ^1^ | 4274 |
| RS-II ^1^ | 2123 |
| SardiNIA | 4694 |
| SHIP | 4067 |
| SOCCS | 1105 |
| Sorbs | 896 |
| TwinsUK | 3640 |
| Young Finns Study | 2023 |
|  |  |
| *Replication cohorts* |  |
| EPIC cases | 793 |
| GHS I | 2995 |
| GHS II | 1179 |
| GSK cases ^1^ | 819 |
| GSK controls ^1^ | 851 |
| Hunter Community Study | 1088 |
| HYPEST | 751 |
| KORA S2 | 3685 |
| Lifelines replication | 5031 |
| LURIC replication GZ | 804 |
| LURIC replication HD | 1156 |
| MARS cases | 636 |
| OGP (Ogliastra) | 9556 |
| OGP-Talana | 1039 |
| SAPALDIA asthmatics | 570 |
| SAPALDIA non-asthmatics | 874 |
| SHIP-Trend | 986 |
| Total | 143160 |

^1^ Studies that are also present in the IGAP sample (see supplemental table 5 for further details)

For all cohort abbreviations, please refer to original study publications.

**Supplemental table 4: Cohorts and participant numbers (for samples of Europeans only) included in the candidate analysis of ascorbate (9), and GWAS of circulating beta-carotene (10), and retinol (11).**

| **Ascorbate** | |  | **β-carotene ^1^** | |  | **Retinol** | |
| --- | --- | --- | --- | --- | --- | --- | --- |
| **Cohorts** | **N** |  | **Cohorts** | **N** |  | **Cohorts** | **N** |
| *Discovery sample* |  |  | InCHIANTI | 1191 |  | *Discovery sample* |  |
| BWHHS | 3425 |  | WHAS I | 404 |  | ATBC | 4014 |
| *Replication* |  |  | WHAS II | 211 |  | PLCO | 992 |
| EPIC-Norfolk | 4501 |  | ATBC | 2126 |  | *Replication* |  |
| MIDSPAN Family gen. 2 | 1814 |  |  |  |  | NHS | 2772 |
| Ten Towns | 1359 |  |  |  |  | InCHIANTI | 1124 |
| BRHS | 3740 |  |  |  |  |  |  |
|  |  |  |  |  |  |  |  |
| *Total* | 148939 |  | *Total* | 3932 |  | *Total* | 8902 |

^1^ In addition to the GWAS including these samples, our analysis also utilised variant-beta-carotene association data from a *BCMO1* fine-mapping study, including data on 4135 participants of the NHS cohort.(12)

For all cohort abbreviations, please refer to original study publications.

**Supplemental table 5: Presumed overlap between samples used in the consortia for GWAS of AD and urate.**

|  | **Number of participants** | |
| --- | --- | --- |
| **Cohorts** | **AD (IGAP consoritum)** | **Urate consortium** |
| AGES-RS | 2772 | 3219 |
| CHS | 2255 | 3252 |
| FHS | 3334 | 7699 |
| KORA F4 | 434 | 1814 |
| *Total* | 8795 | 15984 |
| Proportion of each consortium samples provided by these cohorts (%) | 16.2 | 11.2 |

**Supplemental table 6: Pairwise correlation coefficients between SNPs used to instrument beta-carotene in analyses**

| *SNP* | **rs12934922** | **rs7501331** | **rs4889286** |
| --- | --- | --- | --- |
| **rs12934922** | - |  |  |
| **rs7501331** | -0.28 | - |  |
| **rs4889286** | -0.10 | 0.01 | - |
| **rs4448930** | -0.09 | 0.32 | 0.02 |

SNP correlations were derived from reference data on participants of European ancestry in the 1000 Genomes project, phase 3 (13).

**Supplemental table 7: Details of GWAS datasets used to test associations of antioxidant-instrumenting SNPs with potential confounders / other determinants of AD risk**

| **Trait** | **Reference** | **N** | **Age range (years)** | **Ethnicity** | **Antioxidant SNPs available in datasets** | | | |
| --- | --- | --- | --- | --- | --- | --- | --- | --- |
|  |  |  |  |  | *ascorbate* | *β-carotene* | *retinol* | *urate* |
| Years of education completed | Okbay *et al* (14) | 293723 | 30+ | European | 1 | 4 | 1 | 30 |
|  |  |  |  |  |  |  |  |  |
| Smoking initiation (ever vs. never being a regular smoker) | TAG (Furberg *et al)* (15) | 41969/32066 | 39.6 - 72.3 ^1^ | European | 0 ^2^ | 3 (1 proxy used) ^3^ | 1 | 30 |
|  |  |  |  |  |  |  |  |  |
| BMI | Locke *et al* (16) | 322154 | 12.0 - 107.0 | European | 0 | 3 (1 proxy used) ^3^ | 1 | 30 |
| Triglycerides | Kettunen *et al* (17) | 21544 | 23.9 - 60.9 | European/Erasmus Rupchen | 1 | 4 | 1 | 30 |
| LDL-c | Kettunen *et al* (17) | 21559 | " | " | " | " | " | " |
| Fasting glucose | Kettunen *et al* (17) | 24679 | " | " | " | " | " | " |
| Fasting insulin | Manning *et al* (18) | 51750 | - ^4^ | European | 0 | 2 (1 proxy used) ^5^ | 1 | 30 |

TAG – Tobacco and Genetics Consortium

^1^ range of mean ages across consortium's studies

^2^ The SNP used to instrument ascorbate is a relatively rare variant (minor allele frequency ~ 0.04), and so genotype data for it were not present in several of the GWAS datasets used in these analyses

^3^ rs4448930 missing and no suitable proxy found in these outcome datasets. rs4889286 was missing, but proxy rs6564851 was used instead

^4^ Age of participants not specified in the insulin GWAS article

^5^ rs12934922 lost due harmonisation issue (SNP is palindromic). rs4448930 was missing and no suitable proxy was found in this outcome dataset. Proxy rs6564851 used instead of missing rs4889286.

**Supplemental table 8: Estimates of AD risk according to urate variation, estimated by multiple MR methods ^1^**

|  | Odds Ratio | 95%CI | Heterogeneity test (Q) | Heterogeneity  *P* value | MR-Egger NOME test (%) ^2^ | MR-Egger  Intercept | *P* for intercept test |
| --- | --- | --- | --- | --- | --- | --- | --- |
| **Fixed-effects IVW** | 1.03 | (0.96, 1.10) | 28.7 | 0.48 |  |  |  |
| **Fixed-effects max. likelihood** | 1.03 | (0.96, 1.10) | 28.7 | 0.48 |  |  |  |
| **Weighted median** | 1.04 | (0.95, 1.13) | - | - |  |  |  |
| **Weighted mode** | 1.04 | (0.96, 1.14) | - | - |  |  |  |
| **MR-Egger** | 1.07 | (0.97, 1.19) | 27.1 | 0.51 | 99.4 | -0.005 | 0.22 |

^1^ Median, mode and MR-Egger methods were not applied for other antioxidant analyses because these models require multiple instrumental variable estimates to be conducted robustly.

^2^ Tests for violation of the ‘no measurement error’ (NOME) assumption, derived from the *I^2^* statistic of the genotype-exposure estimates (referred to as *I^2^*_GX_). A value near 100% indicates that the MR-Egger estimate of an effect of circulating urate exposure on AD is unlikely to have been biased towards the null from regression dilution.(19)

**Supplemental table 9: Calculations to indicate power to identify expected differences in AD risk in main MR models, according to genetically predicted variation in antioxidants**

|  | **Total R^2^ in exposure explained by SNPs used as instruments (converted to %)** | **Power (%) to detect OR of following magnitudes ^1^** | | |
| --- | --- | --- | --- | --- |
|  |  | **0.9** | **0.8** | **0.7** |
| Ascorbate | 0.9 | 18.9 | 62.8 | 95.5 |
| Beta-carotene | 6.1 | 80.3 | 100 | 100 |
| Retinol | 0.5 | 12.4 | 39.9 | 77.8 |
| Urate | 7.0 | 85.3 | 100 | 100 |

^1^ Anticipated power to detect odds ratio for AD per SD increase in exposure (assuming true inverse associations) at alpha = 0.05. Calculations are based on the IGAP sample size of 54,162 and ratio of cases to controls of 1:2.1845.

**Supplemental figure 1: Directed acyclic graph illustrating the Mendelian randomization approach**

*G*

*X*

*(e.g.* antioxidants*)*

*U*

*(e.g.* education

attained*)*

[1]

[3]

[2]

**║**

**║**

*Y*

*(e.g.* Alzheimer’s*)*

Observational studies may have established an association between an exposure [X], such as variation in a circulating antioxidant, and outcome [Y], such as risk of Alzheimer’s disease. These studies will be biased from confounding [U] of the X-Y association that is unmeasured/uncontrolled by statistical models, and possibly other sources of bias such as reverse causation. Mendelian randomization can help to assess whether the exposure is causally related to outcome by using a genetic variant [G] (or several in combination) as an instrumental variable for an exposure. This assumes that the genotypes are robust determinants of the exposure [pathway 1]. Due to the independent assortment of alleles for variants between parents and offspring at conception, genotypes that determine the exposure should not also determine confounding factors, nor should disease status modify the genotype (reverse causation) (20). Therefore, G-Y associations should help to infer a causal relationship of X with Y if instrumental variable assumptions hold. There are potential violations to the framework that can induce direct association of genotypes with outcome independently of the exposure and confounders [pathway 2], or indirectly via confounders [pathway 3]. For example, these could arise from horizontal pleiotropy (variants having multiple effects that are independent of exposure determination), linkage disequilibrium between the instrumenting variant(s) and others which affect other traits, and/or population stratification leading to clustering of variant genotypes and confounding traits.

**Supplemental figure 2: Forest plot of MR estimates for the association of urate with AD**

**
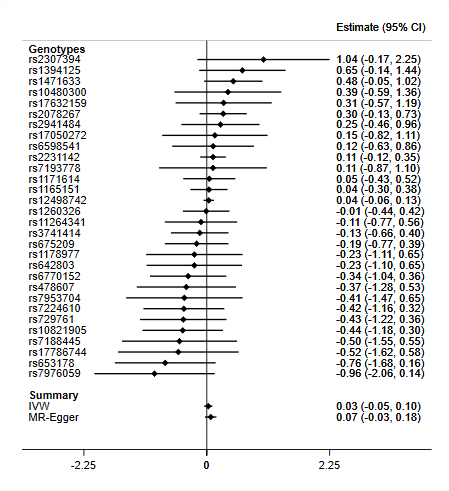
**

Genotypes show Wald estimators for individual SNP-AD associations, with meta-analysis results from the IVW and MR-Egger methods provided under the summary heading. All point estimates and 95% confidence intervals are expressed as log-odds of AD per mg/dL higher urate exposure.

**Supplemental figure 3: Funnel plot of estimates of AD risk for multiple SNPs used to instrument circulating urate**


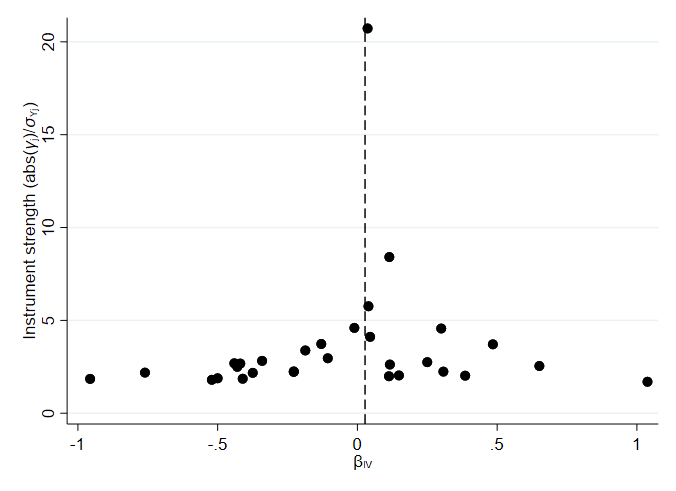


The magnitude of estimated effect of urate on AD from each SNP (X-axis) is plotted against the precision of each of the estimates (Y-axis), with more precise estimates expected to vary less in their estimated magnitudes of effect. A largely symmetric spread of variant estimates in this instance indicates that there is unlikely to be overall directional pleiotropic bias of the results, i.e. any individual pleiotropic effects of variants either toward or away from the null have equalled out.

**References**

1. Greco M, Del F, Minelli C, Sheehan NA, Thompson JR. Detecting pleiotropy in Mendelian randomisation studies with summary data and a continuous outcome. Statistics in medicine. 2015;34(21):2926-40.

2. Bowden J, Davey Smith G, Haycock PC, Burgess S. Consistent estimation in Mendelian randomization with some invalid instruments using a weighted median estimator. Genetic epidemiology. 2016;40(4):304-14.

3. Hartwig FP, Davey Smith G, Bowden J. Robust inference in summary data Mendelian randomization via the zero modal pleiotropy assumption. International Journal of Epidemiology. 2017:dyx102.

4. Bowden J, Davey Smith G, Burgess S. Mendelian randomization with invalid instruments: effect estimation and bias detection through Egger regression. International journal of epidemiology. 2015;44(2):512-25.

5. Burgess S, Bowden J, Fall T, Ingelsson E, Thompson SG. Sensitivity analyses for robust causal inference from mendelian randomization analyses with multiple genetic variants. Epidemiology (Cambridge, Mass). 2017;28(1):30-42.

6. Keenan T, Zhao W, Rasheed A, Ho WK, Malik R, Felix JF, et al. Causal assessment of serum urate levels in cardiometabolic diseases through a Mendelian randomization study. Journal of the American College of Cardiology. 2016;67(4):407-16.

7. Lambert JC, Ibrahim-Verbaas CA, Harold D, Naj AC, Sims R, Bellenguez C, et al. Meta-analysis of 74,046 individuals identifies 11 new susceptibility loci for Alzheimer's disease. Nat Genet. 2013;45(12):1452-8.

8. Kottgen A, Albrecht E, Teumer A, Vitart V, Krumsiek J, Hundertmark C, et al. Genome-wide association analyses identify 18 new loci associated with serum urate concentrations. Nat Genet. 2013;45(2):145-54.

9. Timpson NJ, Forouhi NG, Brion M-J, Harbord RM, Cook DG, Johnson P, et al. Genetic variation at the SLC23A1 locus is associated with circulating concentrations of L-ascorbic acid (vitamin C): evidence from 5 independent studies with> 15,000 participants. The American journal of clinical nutrition. 2010;92(2):375-82.

10. Ferrucci L, Perry JR, Matteini A, Perola M, Tanaka T, Silander K, et al. Common variation in the beta-carotene 15,15'-monooxygenase 1 gene affects circulating levels of carotenoids: a genome-wide association study. Am J Hum Genet. 2009;84(2):123-33.

11. Mondul AM, Yu K, Wheeler W, Zhang H, Weinstein SJ, Major JM, et al. Genome-wide association study of circulating retinol levels. Hum Mol Genet. 2011;20(23):4724-31.

12. Hendrickson SJ, Hazra A, Chen C, Eliassen AH, Kraft P, Rosner BA, et al. beta-Carotene 15,15'-monooxygenase 1 single nucleotide polymorphisms in relation to plasma carotenoid and retinol concentrations in women of European descent. Am J Clin Nutr. 2012;96(6):1379-89.

13. The Genomes Project C. A global reference for human genetic variation. Nature. 2015;526(7571):68-74.

14. Okbay A, Beauchamp JP, Fontana MA, Lee JJ, Pers TH, Rietveld CA, et al. Genome-wide association study identifies 74 loci associated with educational attainment. Nature. 2016;533(7604):539.

15. Furberg H, Kim Y, Dackor J, Boerwinkle E, Franceschini N, Ardissino D, et al. Genome-wide meta-analyses identify multiple loci associated with smoking behavior. Nature genetics. 2010;42(5):441.

16. Locke AE, Kahali B, Berndt SI, Justice AE, Pers TH, Day FR, et al. Genetic studies of body mass index yield new insights for obesity biology. Nature. 2015;518(7538):197.

17. Kettunen J, Demirkan A, Würtz P, Draisma HH, Haller T, Rawal R, et al. Genome-wide study for circulating metabolites identifies 62 loci and reveals novel systemic effects of LPA. Nature communications. 2016;7:11122.

18. Manning AK, Hivert M-F, Scott RA, Grimsby JL, Bouatia-Naji N, Chen H, et al. A genome-wide approach accounting for body mass index identifies genetic variants influencing fasting glycemic traits and insulin resistance. Nature genetics. 2012;44(6):659.

19. Bowden J, Del Greco M F, Minelli C, Davey Smith G, Sheehan NA, Thompson JR. Assessing the suitability of summary data for two-sample Mendelian randomization analyses using MR-Egger regression: the role of the I2 statistic. International Journal of Epidemiology. 2016;45(6):1961-74.

20. Lawlor DA, Harbord RM, Sterne JA, Timpson N, Davey Smith G. Mendelian randomization: using genes as instruments for making causal inferences in epidemiology. Statistics in medicine. 2008;27(8):1133-63.
